# Root morphological and physiological traits and arbuscular mycorrhizal fungi shape phosphorus-acquisition strategies of 12 vegetable species

**DOI:** 10.3389/fpls.2023.1150832

**Published:** 2023-05-08

**Authors:** Zitian Pu, Ruifang Zhang, Hong Wang, Qingyun Li, Jianheng Zhang, Xin-Xin Wang

**Affiliations:** ^1^ State Key Laboratory of North China Crop Improvement and Regulation, Hebei Agricultural University, Baoding, China; ^2^ Mountain Area Research Institute, Hebei Agricultural University, Baoding, China; ^3^ College of Horticulture, Hebei Agricultural University, Baoding, China; ^4^ Key Laboratory of North China Water-Saving Agriculture of Ministry of Agriculture and Rural Affairs, Hebei Agricultural University, Baoding, China

**Keywords:** interspecific variation, intraspecific variation, phosphorus acquisition, root functional traits, root plasticity

## Abstract

Trait plasticity and integration mediate vegetable adaptive strategies. However, it is unclear how patterns of vegetables in root traits influence vegetable adaptation to different phosphorus (P) levels. Nine root traits and six shoot traits were investigated in 12 vegetable species cultivated in a greenhouse with low and high P supplies to identify distinct adaptive mechanisms in relation to P acquisition (40 and 200 P mg kg^-1^ as KH_2_PO_4_). At the low P level, a series of negative correlations among root morphology, exudates and mycorrhizal colonization, and different types of root functional properties (root morphology, exudates and mycorrhizal colonization) respond differently to soil P levels among vegetable species. non-mycorrhizal plants showed relatively stable root traits as compared to solanaceae plants that showed more altered root morphologies and structural traits. At the low P level, the correlation between root traits of vegetable crops was enhanced. It was also found in vegetables that low P supply enhances the correlation of morphological structure while high P supply enhances the root exudation and the correlation between mycorrhizal colonization and root traits. Root exudation combined with root morphology and mycorrhizal symbiosis to observe P acquisition strategies in different root functions. Vegetables respond highly under different P conditions by enhancing the correlation of root traits. Low P supply could significantly improve the direct and indirect ways of mycorrhizal vegetable crops’ root traits axis on shoot biomass, and enhance the direct way of non-mycorrhizal vegetable crops’ root traits axis and reduce the indirect way of root exudates.

## Introduction

Phosphorus (P) is one of the major factors limiting primary productivity in natural and agricultural ecosystems (Vitousek et al., 2010; Johnston et al., 2014), because of its low availability and mobility in most soils (Schachtman et al., 1998). In response to P stress, plants have modulated various root mechanisms to facilitate P uptake (Lambers et al., 2008; Shen et al., 2011; Lizbeth Lopez-Arredondo et al., 2014). Typical root morphological changes include increasing specific root length (SRL) and fine root length ratio to simultaneously broaden the detection range of soil volume by roots (Vance et al., 2003; Postma et al., 2014; Haling et al., 2018). Plants can also mine P by raising organic acid anions, phosphatase, and proton secretion (together known as P-activated exudates) to increase the activation of insoluble inorganic P and organic P in the rhizosphere (Hinsinger et al., 2003; Richardson et al., 2011; Pang et al., 2018). Different P acquisition abilities improves the uptake of soil P such as (1) the increase of root/shoot ratio (RSR) and root length; (2) increased SRL and decreased root tissue density (RTD) (Jonathan, 2018); (3) increased phosphatase activity and carboxylates exudation, and changed the pH of rhizosphere soil, thus increasing the availability of P in the rhizosphere soil (Lambers et al., 2006; Jones et al., 2009; Wang et al., 2020). Plants can develop the ability to adapt low P environment through different combinations of root traits, some plants show changes in root morphological traits, and some enhance the secretion capacity of activated P compounds in roots (Li et al., 2019; Wen et al., 2019). Such variations in the combination of root properties were found in distinct plant species and variants of the same species (Comas and Eissenstat, 2009; McCormack et al., 2015; Weemstra et al., 2016), As a result, plants exhibit varying ability to acquire soil P resources (Gu et al., 2011).

Furthermore, P stress can stimulate and accelerate the creation of symbiotic relationships between plant roots and arbuscular mycorrhizal fungi (AMF), which create symbioses with the majority of plant species and accelerate P uptake (Smith and Read, 2008). Outside of the root P depletion zone, the mycelial network can access inorganic P sources (Clark and Zeto, 2000; Smith et al., 2011; van der Heijden et al., 2015). The adaptive regulation of plant root morphology, P-activated exudates and mycorrhizal symbiosis is beneficial to P uptake by plants. Different mechanisms of P uptake among different strategies are existed significantly that may also be different on the basis of input costs (such as resources and energy) and advantageous to roots for every strategy (Lambers et al., 2006; Ryan et al., 2012; Lynch, 2015). The root morphology of grasses like maize and wheat demonstrated a good adaptive response to P stress (Deng et al., 2014; Lyu et al., 2016; Wen et al., 2017). Increasing the rate of P application can dramatically lower the occurrence of AMF infection in maize and wheat (Teng et al., 2013; Deng et al., 2014; Deng et al., 2017). Meanwhile, legume plants showed a limited morphological response to P supply alterations, whereas root exudates showed considerable modifications (Liu et al., 2016; Lyu et al., 2016; Zhang et al., 2016). Till date, no research has been conducted on the root trait, root exudates, and mycorrhizal colonization of vegetable crops under different P levels. As a result, an intriguing and unanswered scientific question arises: how do vegetable crops coordinate root function features (i.e., root morphology, root exudates, and mycorrhizal traits) in response to changes in soil P availability?

Some of the early studies provide tremendous information by comparing the responses of root systems of different crop species to P supply information. In comparison of root physiology, certain plants demonstrated a good adaptive response to P stress (Deng et al., 2014; Lyu et al., 2016; Wen et al., 2017); similarly, solanaceae showed strong root variation ability, broad bean (*Vicia faba* L.) had limited root morphological responses to varied P supplies, but there were significant alterations in root secretions (Liu et al., 2016; Lyu et al., 2016; Wen et al., 2017). However, root secretions only had a minor inhibitory effect on broad bean roots (Tang et al., 2016). Meanwhile, the recent research has revealed that wheat can improve the plant’s low P tolerance by enhancing the association between above- and below-ground traits under low P conditions, several crops can maximize fitness by enhancing co-variation among root features associated to P acquisition (Wang et al., 2023). It is hypothesized that the soil itself has a low P content in a low P environment, and it requires physiological changes to promote P absorption. As a result, methods such as enhancing the correlation between plant root characteristic are the embodiment of various vegetable species’ low P tolerance. Mycorrhizal colonization was dramatically reduced as P application rate increased, although only slight restriction in broad bean roots was observed (Teng et al., 2013; Deng et al., 2014; Tang et al., 2016; Deng et al., 2017; Wang et al., 2020). These findings revealed that, in response to low P stress, there could be synergies and trade-offs between root function aspects of different plants, culminating in diverse subsurface methods for improving P acquisition by different crop species. However, most previous research has focused on one or two traits among crop root shape, root secretion, and mycorrhizal symbiosis, with only a few vegetable crop species addressed concurrently (Brown et al., 2013; Deng et al., 2014; Lyu et al., 2016; Wen et al., 2017). As a result, it is necessary to monitor and analyze the response qualities of these root functional traits across a large range of vegetable crop species at the same time. This will lead to a better understanding of the interplay between root characteristics in varied soil environments, ultimately enhancing soil P uptake and the performance of vegetable crops.

The purpose of our study is to address: (1) how do vegetable plants coordinate root functional features (i.e., root morphology, root exudates, and mycorrhizal qualities) in response to variations in external soil P availability? (2) the effects of diverse P environments on the correlation of root characteristics in several vegetable species. This study determined the above- and below-ground features by setting the two components 12 vegetable species at two P levels), selected six genera of different crops, under varied P source environment. We primarily evaluated the two hypotheses listed below: (1) there are trade-offs among root morphology, exudates, and mycorrhizal colonization. different types of root functional characteristics (root morphology, exudates, and mycorrhizal properties) respond differently to variable soil P levels within or between vegetable species; (2) the association between root characteristics of vegetable crops is stronger at lower P availability than at higher P availability.

## Materials and methods

### Experimental design

The experiment was set up as a randomized complete block design, with two factors: (1) vegetable species: 12 vegetable species; (2) P supplies (as KH_2_PO_4_): 40 and 200 mg P kg^-1^ soil. The experiment was carried out with five replicates and total 120 pots were used. To reduce the effects of fixed plant position within blocks, pots within each block were re-randomized weekly.

### Vegetable species and soil collection

We selected 12 common vegetable species, nine of the species form a symbiosis with AMF (Amaryllidaceae: allium (*Allium fistulosum* L., Af), garlic (*Allium sativum* L., As); Cucurbitaceae: cucumber (*Cucumis sativus* L.), melon (*Citrullus lanatus*),; Compositae: lettuce (*Lactuca sativa* Linn.); chrysanthemum (*Chrysanthemum coronarium* L.); Solanaceae: pepper (*Capsicum annuum*), eggplant (*Solanum melongena* L.), tomato (*Lycopersicon esculentum*). Three of the species were well-known non-AM (NM) species, Chenopodiaceae: spinach (*Spinacia oleracea* L.), sugar beet (*Beta vulgaris* L.); cruciferous: rape (*Brassica chinensis* Linn.); These were included in the experiment based on their agronomic importance and the fact that their P-acquisition techniques are expected to differ from AM species. The 12 vegetable species show a varied evolutionary lineage and a wide range of variation in root functional properties.

The low-P soil fallow for two years was collected from Sun Gezhuang Village, Xiongxian County, Baoding City, Hebei Province (116°09’ E, 39°05’ N). The 5 mm sieved, air-dried soil was mixed thoroughly. The soil properties were as follows: pH 7.38 (ratio of clay to water was 1:2.5), total nitrogen 1.32 g kg^-1^, available P 2.29 mg kg^-1^, available potassium 123 mg kg^-1^, organic carbon 13.6 g kg^-1^, and total potassium 19.73 g kg^-1^. Plastic flowerpots (height: 15 cm, top diameter: 20 cm, and bottom diameter: 12 cm) were filled with 2 kg dry soil per pot.

We used a P supply of 40 mgkg^-1^ soil for the low-P treatment (LP), and 200 mgkg^-1^ soil for the high-P treatment (HP), KH_2_PO_4_ was used as P resource. soil was also supplemented with basal nutrients at the following rates (mg kg^-1^): N 100 (NH_4_)_2_SO_4_), K 327 (K_2_SO_4_, KH_2_PO_4_), Ca 46 (CaCl_2_), Mg 4.2 (MgSO_4_7H_2_O), Fe 0.88 (EDTAFe-Na), Na 0.36 (EDTAFe-Na), Mn 1.8 (MnSO_4_H_2_O), Zn 2.5 (ZnSO_4_7H_2_O), Cu 0.57 (CuSO_4_5H_2_O) to optimize the required nutrient availability. To maintain the same soil K level among all treatments, K_2_SO_4_ was supplied at 561 mg K kg^−1^ soil in the treatments at 40 mg.kg^-1^ soil. The indigenous AMF colonized the roots of crops cultivated in the native soil, no extra AMF inocula were added.

### Plant growth conditions

The experiment was conducted in a light culture room at the west campus of Hebei Agricultural University, Baoding City, Hebei Province (39°04’ N, 116°09’ E). Seeds were surface-sterilized (30 min in a 5.0% (v/v) H_2_O_2_ solution), rinsed, and germinated in a growth chamber with a dark and humid environment at 20°C. Depending on species, three to six uniform seedlings were planted per pot, and seedlings were later thinned to the plant number of one. Pots were watered daily by weight to 60% ± 10% field capacity. The room temperatures were maintained at 22-27°C with relative humidity of 45%-55%. All crop varieties begin to grow on July 21st, 2019. Twelve vegetables were harvested on September 15th, 2019, and the remaining two allium vegetables were harvested on October 1st, 2019, because of their initial slow growth of allium. At time of harvest, the visual differences in shoot growth of all 12 vegetables were observed under both LP and HP conditions.

### Harvest and measurements

At the time of harvest, the leaves and stem were separated from the root system with scissors. The fresh biomass of the above-ground was measured and stored into the envelope to keep into the oven, which was dried at 105°C for 30 min and 72°C for 48 h. Shoot P concentration was determined by the standard vanado-molybdate method (Murphy and Riley, 1986) after digestion in a H_2_SO_4_-H_2_O_2_ mixture at 360°C for 2 h. We measured ten root traits in three categories: seven root morphological traits: root biomass, root/shoot ratio, root length and fine roots (root diameter < 0.2 mm) length, root diameter, SRL and specific fine root length (SFRL); and two root physiological traits: carboxylates in the rhizosphere, acid phosphatase activity in the rhizosphere; and mycorrhizal colonization. A full description of every root trait is explained as below.

After sampling the rhizosphere soil, all visible roots were picked out from every pot. Root samples were washed with de-ionized water and frozen at -20°C prior to measurement of root morphological parameters. Cleaned root samples were dispersed in water in a transparent array (30 × 20 × 3 cm) and scanned with an EPSON scanner at a resolution of 400 dpi (Epson Expression 1600 pro, Model EU-35, Japan). The root traits such as length and diameter were determined by analysis of images using WinRHIZO Pro software (Regent Instruments Inc, Quebec, Canada) software. SRL (m g^-1^) was assessed as the ratio of root length over root weight. After scanning of root samples, the roots were also oven-dried at 70°C for three days and weighed as root biomass to further calculating the root/shoot biomass ratio. In addition, we calculated SRL by root length over root biomass, assuming that roots were perfectly cylinders.

Acid phosphatase activity in the rhizosphere was measured according to (Alvey et al., 2001) using *p*-nitrophenylphosphate (*p*-NPP). The roots with tightly adhering rhizosphere soil were transferred into 200-mL vials containing 0.2 mM CaCl_2_ solution depending on root volume (Murphy and Riley, 1986). The pH value of Na-acetate buffer (200 mM) was adjusted to the average pH values (5.4) of the rhizosphere soil. The rhizosphere soil in the CaCl_2_ suspension was separated by centrifugation of 10 min at 12,000 × *g* and dried at 60°C before weighing it. The concentration of *p*-NPP in the supernatant was measured spectrophotometrically at 405 nm.

Carboxylates in the rhizosphere soil were analyzed using a reversed phase high performance liquid chromatography (HPLC) system according to a previous report (modified from Shen et al., 2003). The chromatographic separation was conducted on a 250 × 4.6 mM reversed-phase column (Alltima C18, 5 Micrometers; Alltech Associates Inc., Deerfield, IL, USA). The mobile phase was 25 mM KH_2_PO_4_ (pH 2.25) with a flow rate of 1 ml min^-1^ at 31°C. Detection of carboxylates was carried out at 214 nm (Zhang et al., 2019).

Root samples were treated with 10% (w/v) KOH solution in a 90°C water bath for 20 min, rinsed with water, acidified with 2% (v/v) HCl for 5 min at room temperature to make it transparent, and then stained with 0.05% (w/v) nonvital Trypan blue in a 90°C water bath for 30 minutes. Stained root fragments were placed in a lactic acid-glycerol-water (v/v/v, 1:1:1) solution overnight to remove excess stain. For each sample, 30 stained root fragments of second-order roots with an average length of 1 cm were selected randomly and observed with light microscope after mounted on two slides. Colonization by AMF (%) was assessed using the method described by Trouvelot et al. (1986).

### Data analysis

To find out how two shoot parameters and eleven root features of 12 distinct vegetable species responded to varied P levels, we used a two-way ANOVA with a randomized block to examine the effects of P treatments, two main factors and their interaction on shoot biomass, P concentration, and eleven root traits. Significant differences among means were based on the t-test (*P* < 0.05). Spearman’s correlation analysis was used to investigate the pairwise associations of root traits in order to examine possible coordination or trade-offs in variance of the two shoot parameters and eleven root traits among 12 vegetable traits. All statistical analyses were performed with the SPSS 26.0 statistical software (IBM Corp., Armonk, NY., USA).

We also used principal component analysis (PCA) to investigate trait interactions and to represent vegetable species in a lower dimensional space. PCA plots were constructed in Origin 2021b (OriginLab, Northampton, MA., USA).

We used linear regression to investigate the associations between root trait, AMF, A-Pase, and carboxylates and shoot biomass. We anticipated that the first root trait axis would contribute to shoot biomass both directly and indirectly through its interactions with AMF, A-Pase, and carboxylates. We focused on the root trait axis rather than individual root traits because we lacked the statistical power to fit causal linkages between root traits, AMF, A-Pase, and carboxylates while meeting model fit parameter criteria.

## Results

### Shoot trait variations in response to P concentration

**Figure 1:**
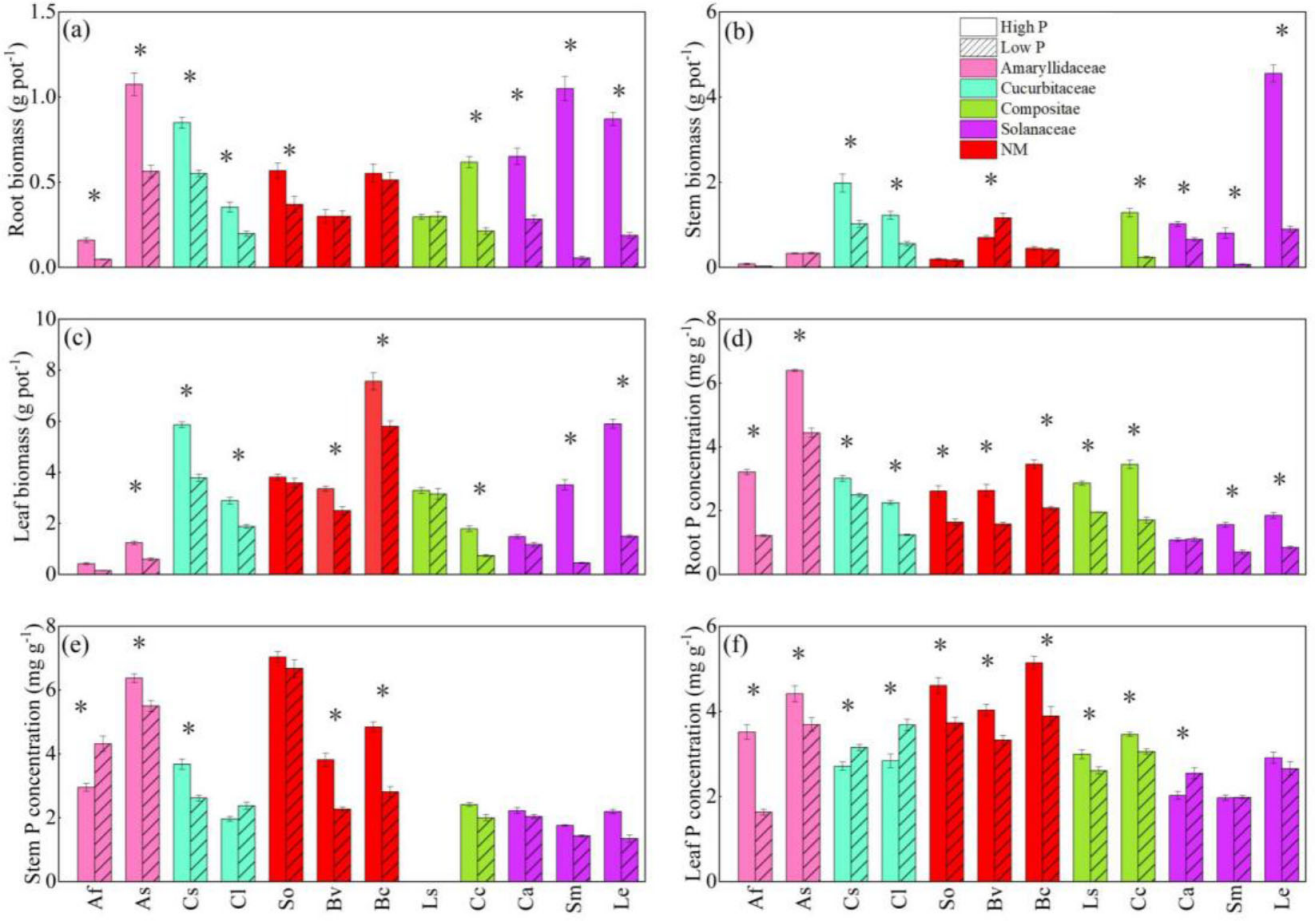
The responses of root biomass **(A)**, stem biomass **(B)**, leaf biomass **(C)**, root P concentration **(D)**, stem P concentration **(E)**, and leaf P concentration **(F)** to soil phosphorus **(P)** availability among 12 crop species. Each value is the mean ( ± SE) of fifive replicates. * indicates significant difference between different P treatments within each species (based on Tukey’s *post hoc* analysis, *P* ≤ 0.05). Species abbreviation: allium (*Allium fistulosum* L., Af); garlic (*Allium sativum* L., As); cucumber (*Cucumis sativus* L., Cs); melon (*Citrullus lanatus*, Cl); spinach (*Spinacia oleracea* L., So); sugar beet (*Beta vulgaris* L., Bv); rape (*Brassica chinensis* Linn., Bc); lettuce (*Lactuca sativa* Linn., Ls); chrysanthemum (*Chrysanthemum coronarium* L., Cc); pepper (*Capsicum annuum*, Ca); eggplant (*Solanum melongena* L., Sm); tomato (*Lycopersicon esculentum*, Le).

The two shoot features showed significant variance among species, as demonstrated by the two-way ANOVA (**Table 1**). The interaction of P applications and species had a significant effect on root biomass and root P concentration (*P* < 0.05). Low P treatment reduced the biomass of all parts of vegetables and the variation of different P application in all parts of vegetables under low P condition is consistent with the variation of biomass (**Figures 1A–F**). At the same time, the biomass and P concentration of fruit vegetables were more significantly responsive to P level changes than that of leaf vegetables. For example, which the change of solanaceae was obvious, compared with high P treatment, root, stem and leaf biomass of eggplant was reduced by 94.7%, 91.1%, and 85.2%, respectively (**Figures 1A–C**). Meanwhile, the P concentration in different parts of other vegetable crops decreased significantly except that the P concentration in stem of scallion, watermelon, and leaf of pepper increased significantly. For example, stem P concentration in onion increased 46.2% (**Figure 1E**). Cucumber and watermelon leaf P concentration increased 16.2% and 29.8%, respectively (**Figure 1F**). The P concentration of non-mycorrhizal plants decreased obviously (**Figures 1D–F**).

**Table 1:** Results of two-way ANOVA for root functional traits among 12 vegetables species and two soil phosphorus (P) treatments.

Traits	Species			P treatment			Species ×P treatment		
	df	F	*P* value	df	F	*P* value	df	F	*P* value
ShB	11	268	< 0.001	1	638	< 0.001	11	75.6	< 0.001
RB	11	57.4	< 0.001	1	443	< 0.001	11	34.3	< 0.001
SPC	11	367	< 0.001	1	217	< 0.001	11	28.3	< 0.001
RPC	11	304	< 0.001	1	870	< 0.001	11	19.7	< 0.001
RD	11	35.0	< 0.001	1	64.1	< 0.001	11	4.24	< 0.001
SFRL	11	16.4	< 0.001	1	38.6	< 0.001	11	8.72	< 0.001
SRL	11	15.7	< 0.001	1	33.8	< 0.001	11	7.62	< 0.001
FRL	11	38.5	< 0.001	1	122	< 0.001	11	16.6	< 0.001
RL	11	127	< 0.001	1	456	< 0.001	11	67.5	< 0.001
RTD	11	13.4	< 0.001	1	1.62	0.206	11	4.15	< 0.001
A-Pase	11	21.8	< 0.001	1	3.24	0.075	11	4.29	< 0.001
Carboxylates	11	154	< 0.001	1	119	< 0.001	11	20.5	< 0.001
MC	8	213	< 0.001	1	547	< 0.001	8	18.3	< 0.001

The data in (MC) are without non-mycorrhizal species (n=10). Trait abbreviations: Shoot biomass (ShB), root biomass (RB), shoot P concentration (SPC), root P concentration (RPC), average diameter of roots (RD), specific fine root length (SFRL), specific root length (SRL), fine root length (FRL), root length (RL), root tissue density (RTD), colonization by arbuscular mycorrhizal fungi (MC), the amounts of carboxylates in the rhizosphere (carboxylates) and acid phosphatase activity in the rhizosphere (A-Pase).

### Root trait variations in response to P concentration

**Figure 2:**
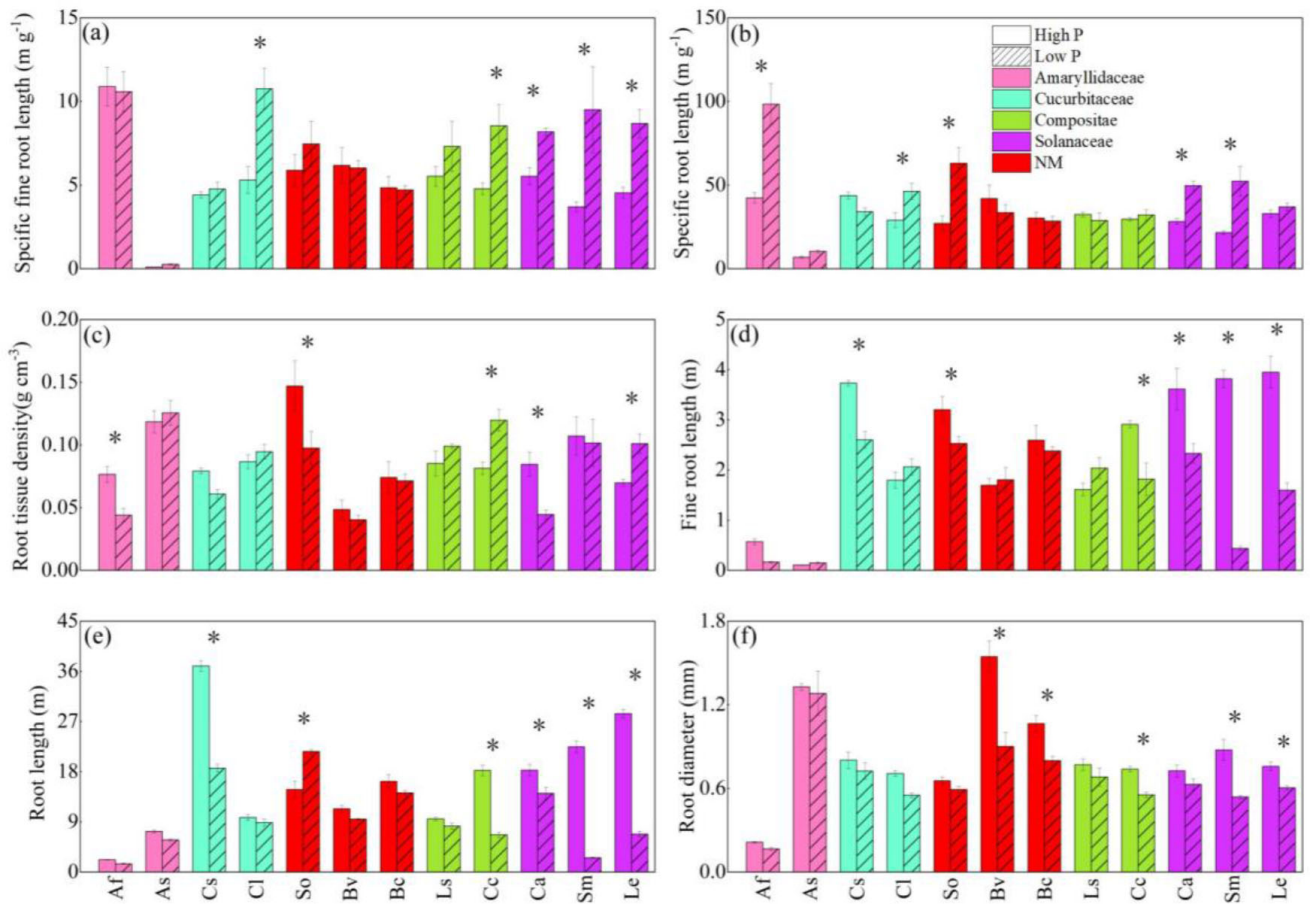
The responses of specific fine root length **(A)**, specific root length **(B)**, root tissue density **(C)**, fine root length **(D)**, root length **(E)**, and average diameter of roots **(F)** to soil phosphorus **(P)** availability among 12 crop species. Each value is the mean (± SE) of five replicates. * indicates significant difference between different P treatments within each species (based on Tukey’s *post hoc* analysis, *P* ≤ 0.05). Species abbreviation: allium (*Allium fistulosum* L., Af); garlic (*Allium sativum* L., As); cucumber (*Cucumis sativus* L., Cs); melon (*Citrullus lanatus*, Cl); spinach (*Spinacia oleracea* L., So); sugar beet (*Beta vulgaris* L., Bv); rape (*Brassica chinensis* Linn., Bc); lettuce (*Lactuca sativa* Linn., Ls);chrysanthemum (*Chrysanthemum coronarium* L., Cc); pepper (*Capsicum annuum*, Ca); eggplant (*Solanum melongena* L., Sm); tomato (*Lycopersicon esculentum*, Le).

The eleven root functional properties varied substantially among species (**Table 1**). Eleven traits also varied significantly with the interaction of P treatment and species (*P* < 0.05). As a result, the intensity and direction of root trait responses to P supply in different soils diverged between species. All eleven traits varied greatly among all 12 vegetables at two P levels (**Figures 2A–F**). At the same time, the root structure and root exudates of fruit vegetable species were more sensitive to phosphorus water than those of leaf vegetable species (**Figures 2A–F**). Under the LP condition, SFRL of two solanaceae vegetables, watermelon and chrysanthemum was significantly enhanced regardless of low or high P application. Eggplant showed the prominent noticeable variation, increasing by 61.1% (**Figure 2A**). SRL of onion, watermelon, solanaceae plants and spinach were significantly increased under low P condition, compared with the traits under the HP condition. Garlic showed the largest increase by 121% (**Figure 2B**). RTD of onion, chrysanthemum, pepper, spinach and carrot non-mycorrhizal plants were severely altered by LP except for garden chrysanthemum, RTD of other plants was significantly decreased (**Figure 2C**).

Compared with the three root structural traits, the morphological traits of each vegetable root altered more significantly (**Figure 2**). Under the LP condition, cucumber, spinach, chrysanthemum, pepper and eggplant all showed similar variation of fine root length and root length, which were lower than at HP treatment (**Figures 2D, E**). The two root morphological traits of eggplant reduced more significantly significant (**Figures 2D, E**). Compared with HP, fine root length and root length of eggplant at LP were reduced 75.9%, 91.4%, respectively (**Figures 2D, E**).

**Figure 3:**
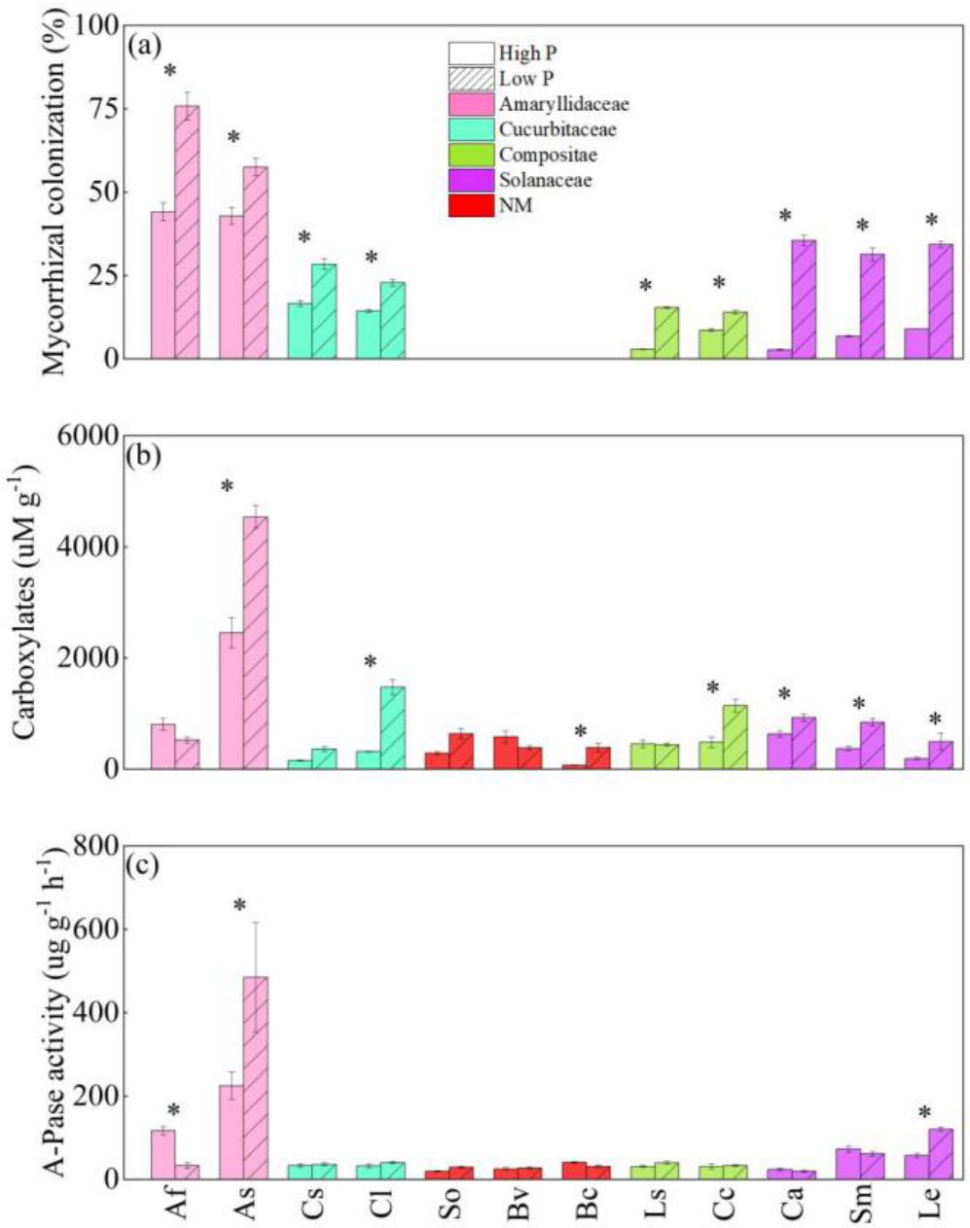
The responses of mycorrhizal colonization **(A)**, the amounts of carboxylates in the rhizosphere (carboxylates) **(B)**, and acid phosphatase activity in the rhizosphere (A-Pase) **(C)** to soil phosphorus **(P)** availability among 18 crop species. Each value is the mean ( ± SE) of fifive replicates. * indicates significant difference between different P treatments within each species (based on Tukey’s *post hoc* analysis, *P* ≤ 0.05). Species abbreviation: allium (*Allium fistulosum* L., Af); garlic (*Allium sativum* L., As); cucumber (*Cucumis sativus* L., Cs); melon (*Citrullus lanatus*, Cl); spinach (*Spinacia oleracea* L., So); sugar beet (*Beta vulgaris* L., Bv); rape (*Brassica chinensis* Linn., Bc); lettuce (*Lactuca sativa* Linn., Ls); chrysanthemum (*Chrysanthemum coronarium* L., Cc); pepper (*Capsicum annuum*, Ca); eggplant (*Solanum melongena* L., Sm); tomato (*Lycopersicon esculentum*, Le).

LP treatment significantly enhanced mycorrhizal colonization (**Figure 3A**) for all 10 AM vegetable species, especially for solanaceae crops. Mycorrhizal colonization of eggplant and pepper was increased up to 35.6% and 31.3%. Despite the fact that high P treatment reduced AMF colonization in some crops, allium plants had a higher AMF colonization. For example, allium and garlic had 75.8% and 57.5% mycorrhizal colonization at LP, although HP supply considerably hindered mycorrhizal colonization, they maintained high mycorrhizal colonization (44.1% and 42.9%).

HP supply inhibited the content of carboxylates in the rhizosphere (**Figure 3B**). The content of carboxylates in the rhizosphere of vegetables was significantly increased (46.4% ~ 457.1%) under the LP condition compared to HP treatment. Both at LP and HP level, only A-Pase activity of onion and garlic was significantly varied (**Figure 3C**), and LP levels reduced onion A-Pase activity considerably while increasing the A-Pase activity of garlic.

### Correlations between shoot P concentration and root traits among 12 vegetables varieties

As indicated in **Table 2**, the LP treatment considerably increased the correlations between root features when compared to the HP treatment. Mycorrhizal colonization was substantially correlated with SRL and SFRL in the LP condition. Under the LP condition, RTD was substantially associated with mycorrhizal colonization, carboxylaters, and A-Pase as compared to the HP condition.

**Table 2:** Phenotypic correlations coefficients among plant traits across 12 vegetables species grown at the high P level (200 mg P kg^-1^ soil, low-left diagonal) and at the low P level (40 mg P kg^-1^ soil, upper-right diagonal).

	ShB	RB	SPC	RPC	RD	SFRL	SRL	RTD	A-Pase	Carboxylates	MC
ShB		0.65**	-0.01	0.46**	0.48**	-0.28**	-0.29**	-0.14	-0.29*	-0.55**	-0.49**
RB	0.29*		0.20	0.79**	0.78**	-0.64**	-0.66**	0.12	-0.09	-0.13	-0.11
SPC	0.01	0.02		0.22	0.16	0.07	0.05	0.05	-0.09	0.30**	0.41**
RPC	-0.26*	-0.07	0.59**		0.58**	-0.59**	-0.59***	0.12	-0.03	-0.07	-0.15
RD	0.16	0.30*	0.21	0.20		-0.70**	-0.70**	0.02	0.05	-0.18	0.14
SFRL	0.12	-0.52**	-0.07	0.01	-0.24		0.99**	-0.46**	-0.27**	-0.01	0.23
SRL	0.11	-0.52**	-0.07	0.01	-0.24	0.99**		-0.47**	-0.25	0.01	0.23
RTD	-0.16	0.43**	0.06	0.04	-0.14	-0.64**	-0.64**		0.49**	0.37**	-0.35*
A-Pase	-0.16	0.29**	0.03	0.28*	0.15	-0.18	-0.18	0.04		0.27**	0.24
Carboxylates	-0.82**	-0.15	-0.03	0.14	0.04	-0.19	-0.18	0.07	0.25		0.02
MC	0.69**	0.13	-0.28	-0.32*	0.10	0.09	0.09	-0.26	-0.22	-0.62**	

The data in (MC) are without non-mycorrhizal species (n=9). Trait abbreviations: Shoot biomass (ShB), root biomass (RB), shoot P concentration (SPC), root P concentration (RPC), average diameter of roots (RD), specific fine root length (SFRL), specific root length (SRL), root tissue density (RTD), colonization by arbuscular mycorrhizal fungi (MC), the amounts of carboxylates in the rhizosphere (carboxylates) and acid phosphatase activity in the rhizosphere (A-Pase). Significant correlations are in bold: *, 0.01 < P ≤ 0.05; **, P ≤ 0.01.

Under LP condition, 24 groups of root traits were noticeably correlated. But at HP treatment, only 4 groups of root traits were significantly correlated. Compared with the LP treatment, the correlation of root traits of vegetable crops decreased by 83.33% at the HP level.

### Correlations among eight root traits under HP and LP conditions

**Figure 4:**
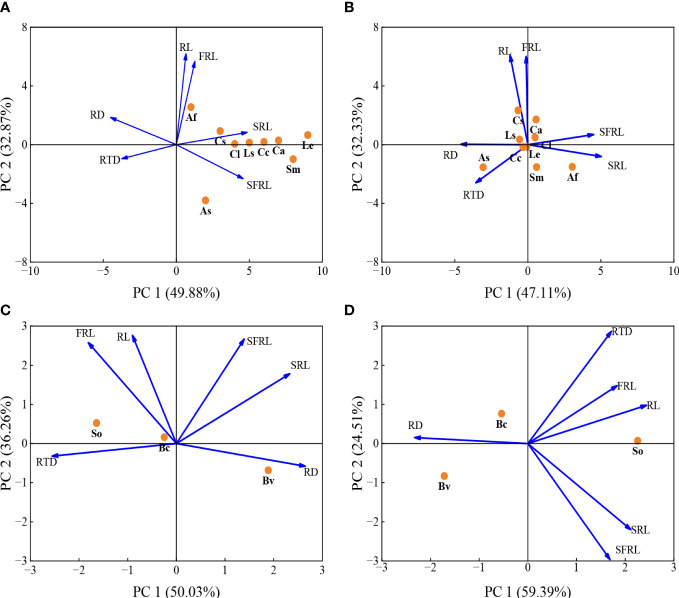
Principal component analysis (PCA) of six root traits for 9 mycorrhizal vegetables species in response to the high P (200 mg kg^-1^ soil applied; **(A)**, the low P supply (40 mg kg^-1^ as KH_2_PO_4_; **(B)** and 3 non-mycorrhizal vegetables species in response to the high P (200 mg kg^-1^ soil applied; **(C)**, the low P supply (40 mg kg^-1^ as KH_2_PO_4_; **(D)**. Trait notations: average diameter of roots (RD), specific fine root length (SFRL), specific root length (SRL) fine root length (FRL), root length (RL), root tissue density (RTD). Species abbreviation: allium (*Allium fistulosum* L., Af); garlic (*Allium sativum* L., As); cucumber (*Cucumis sativus* L., Cs); melon (*Citrullus lanatus*, Cl); spinach (*Spinacia oleracea* L., So); sugar beet (*Beta vulgaris* L., Bv); rape (*Brassica chinensis* Linn., Bc); lettuce (*Lactuca sativa* Linn., Ls); chrysanthemum (*Chrysanthemum coronarium* L., Cc); pepper (*Capsicum annuum*, Ca); eggplant (*Solanum melongena* L., Sm); tomato (*Lycopersicon esculentum*, Le).

PCA results demonstrated that 6 root functional traits of 9 mycorrhizal vegetables species in the HP treatment showed 82.8% variation in the first two components (**Figure 4A** and **Table S1**). At the HP level, the first two principal components (PC) made up 49.9% and 32.9% of the total variation (**Figure 4A**). Root diameter, RTD, SRL and SFRL had a strong contribution to PC1, whereas root length and fine root length mainly contributed to PC2 (**Figure 4A**). Under the LP condition, the first two PCs made up 47.1% and 32.3% of the total variation (**Figure 4B**).

PCA based on 6 root functional traits of 3 non-mycorrhizal vegetables species (**Figures 4C, D**). At the HP level, the first two principal components (PC) made up 50.0% and 32.3% of the total variation (**Figure 4C**). Root diameter, RTD and SRL had a strong contribution to PC1, whereas root length, fine root length and SFRL mainly contributed to PC2 (**Figure 4C**). At the LP level, the first two PCs made up 59.4% and 29.5% of the total variation (**Figure 4D**).

### Pathway analysis of root traits, AMF, A-Pase, carboxylates and shoot biomass

**Figure 5:**
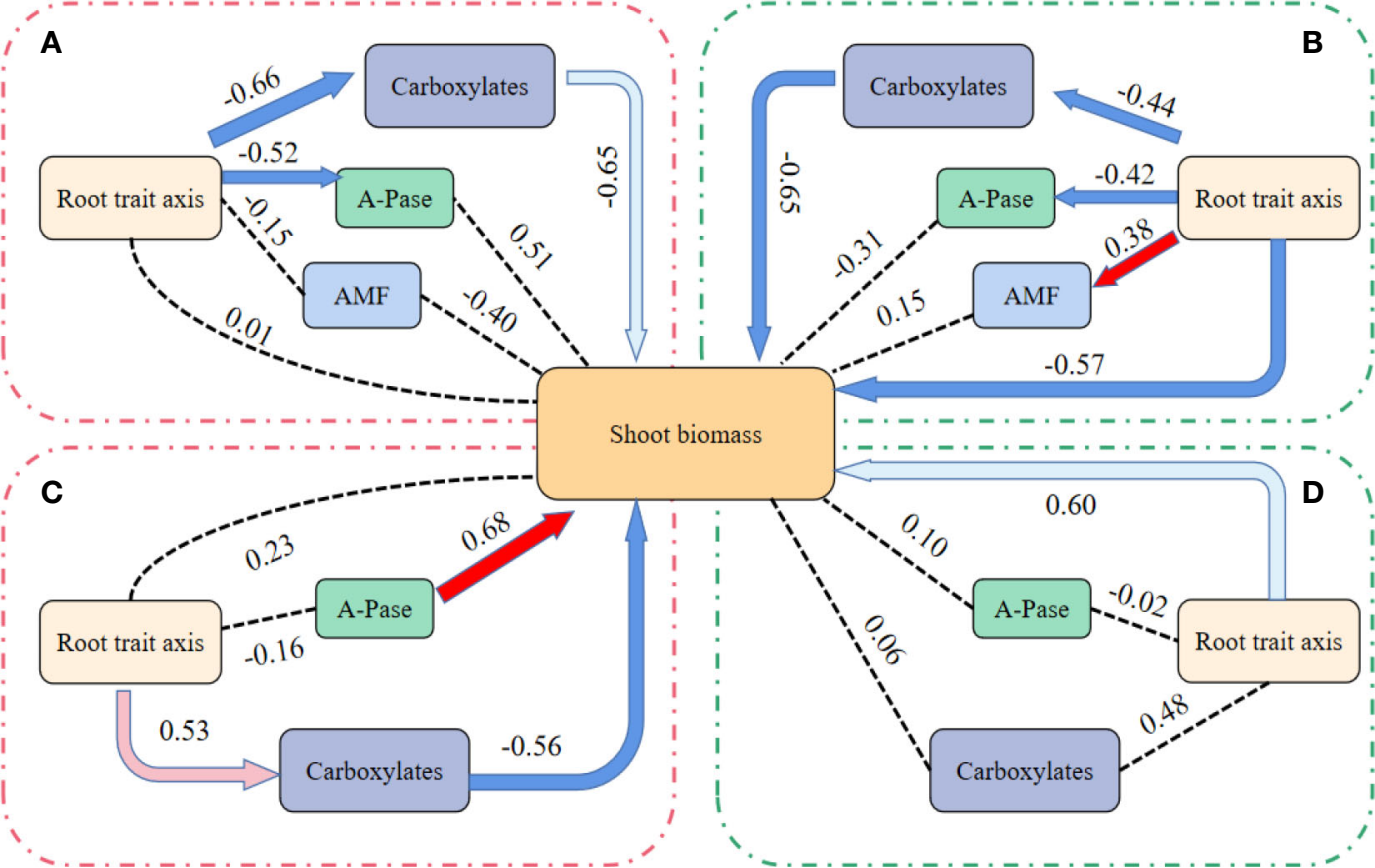
Effects of root traits axis, mycorrhizal colonization, (AMF) the amounts of carboxylates in the rhizosphere (carboxylates) and acid phosphatase activity in the rhizosphere (A-Pase) on shoot biomass. First axis of the root economics traits, AMF, carboxylates and A-Pase in path analysis for mycorrhizal vegetables species to the high P (200 mg kg^-1^ soil applied; **(A)**, the low P supply (40 mg kg^-1^ as KH_2_PO_4_; **(B)** and non-mycorrhizal vegetables species to the high P (200 mg kg^-1^ soil applied; **(C)**, the low P supply (40 mg kg^-1^ as KH_2_PO_4_; **(D)**. Red arrows indicate positive relationship, and blue arrows indicate negative relationship. Dark solid line arrow indicates significant relationships (*P* < 0.01), light-colored line arrow indicates significant relationships (0.01 < *P* < 0.05) and dashed lines indicate that there is no path association (*P* > 0.05).

In the PCA, more vegetables species preferred axis 1, so axis 1 was taken as the root axis (**Figure 4**). Under the HP condition, shoot biomass of mycorrhizal vegetables species was indirectly measured by the first axis of root traits through carboxylaters (**Figure 5A**; RFA; RMSEA < 0.001). In addition, RFA can also direct affect A-Pase activity (path analysis model fit:RMSEA < 0.001). Except for the indirect root axis-carboxylater pathway, the direct root axis pathway was improved under the LP condition (path analysis model fit: RMSEA < 0.001; **Figure 5B**). Meanwhile, RFA can also directly affect A-Pase activity and mycorrhizal colonization (path analysis model fit:RMSEA < 0.001; **Figure 5B**).

Under the HP condition, shoot biomass of non-mycorrhizal vegetables species was measured indirectly *via* the first axis of root traits through carboxylates (**Figure 5C**; RFA; RMSEA < 0.001). In addition, A-Pase activity can also directly affect the shoot biomass (path analysis model fit:RMSEA < 0.001; **Figure 5C**). Under the LP condition, only the direct pathway in the root axis affect shoot biomass (path analysis model fit: RMSEA < 0.001; **Figure 5D**).

## Discussion

### Shoot and root growth traits of 12 vegetable species

P concentration in stem and leaf is not only an important indicator for evaluating P nutrition in plants, but it is also an important method for determining whether P stress inhibits crop yield (Barry and Miller, 1989; Bollons and Barraclough, 1999). In this study, the P concentration in the shoots of 12 vegetables declined at low P level, and the changes in biomass and P concentration in all regions of the vegetables appeared to be constant. Under low P condition, all vegetables showed consistent changes of shoot biomass and P concentration, especially in leaves (**Figure 1**).

Previous researches revealed that the trade-offs between root traits and mycorrhizal colonization in nutrient acquisition play a significant impact in plant P uptake in natural ecosystems (Lambers et al., 2006; Liu et al., 2015; Chen et al., 2016). According to the results, various vegetable root traits and mycorrhizal colonization demonstrated varied mechanisms for soil P uptake under low P conditions (**Figures 2**, **3**). Generally, low soil P availability and shoot P deficiency improve the regulation of root morphology, the release of P activated exudates, or the alternative strategy: the symbiotic interaction with AMF (Ryan et al., 2012), whereas they are significantly inhibited by high shoot P concentration and high soil P availability (Teng et al., 2013; Deng et al., 2017; Wen et al., 2017). All of these adaptive responses indicated that plants may modify numerous root functional properties to achieve a balance of costs and benefits under varied P conditions (Lynch, 2015; van der Heijden et al., 2015; Weemstra et al., 2016). We found that lettuce, chrysanthemum under low P condition had high RTD and low SRL (**Figure 2**). Exploration of bigger soil volumes in low P conditions leads to a reduced cost per unit root length (McCormack et al., 2015; Kramer-Walter et al., 2016).

In this study, spinach, beet and rape were all non-mycorrhizal vegetables (Wang and Qiu, 2006; Lambers et al., 2013), and we did not observe mycorrhizal colonization of these vegetable species. Non-mycorrhizal vegetables had more stable root structure and morphological characteristics (**Figure 2**). Non-mycorrhizal plants did not exhibit stronger root exudation capabilities under low P condition. Most non-mycorrhizal plants adopt a single strategy under low P condition, and non-mycorrhizal plants acquired P by depending on root morphological and structural traits as compared to mycorrhizal vegetable species.

Increasing soil P availability significantly reduced the symbiotic relationship between roots and AMF (Teng et al., 2013). We concluded that increased P levels reduced mycorrhizal colonization in all mycorrhizal plants. It is noteworthy that mycorrhizal colonization of the two alliaceae species were the highest under both low and high P conditions. This result may be due to the shorter root length, finer root length and the smaller root surface area of alliaceae species (**Figure 2**). In addition, a large number of previous studies have reported that alliaceae plants have a relatively thicker root diameter (Itoh and Barber, 1983; Föhse et al., 1991), probably leading to the weak regulation of root morphology in allium plants. As a result, they rely more on mycorrhizal symbiosis to improve the detection of soil P, even under high P conditions (Wen et al., 2019); for instance, the onion plants in the Netherlands showed high colonization under high P conditions (Galvan et al., 2009).

Most vegetable species did not depend prominently on carboxylates and A-Pase to under low P stress to acquire P (**Figures 3B, C**). These results were different from the results of Wen et al. (2019), who found under low P condition, fine-rooted plants usually showed lower carboxylater content and A-Pase activity in root sheath. Meanwhile, studies have shown that both root morphology and mycorrhizal colonization are regulated by P level (Lambers et al., 2008; Shen et al., 2011). The release of P-activated secretions (such as organic acid anions) is a “P mining” strategy, but all of these strategies are modulated in favor of P uptake by plants. However, the input costs determined by each strategy may restrict the ability of vegetables to effectively express all strategies (Lynch and Ho, 2005; Ryan et al., 2012; Raven et al., 2018); as a result, the expression form of vegetable root exudates was not important in this investigation.

The results showed that fruit vegetables were more responsive to changes in different P levels (**Figures 1A–F**, **2A–F**). This may be fruit vegetables have different nutrient requirements compared with leaf vegetables. Leaf vegetables require more N throughout the growth cycle and more P at the peak of growth. However, the requirement of N and P of fruit vegetables was high during the whole growth cycle. Thus, the fruit vegetable species could be more inhibited by low P stress than leaf vegetable species.

### Correlations and pathway relationship of root functional traits among vegetable species

In this study, we found that only a few root traits were related to shoot P concentration in both P treatments (**Table 2**), suggesting that plant P uptake was related to root biomass, root length and RTD (**Table 2**). This result is consistent with the previous study by Kraft et al. (2015). However, the association of each individual trait with shoot growth is based on a complex trait co-variation, especially in the case of P deficiency (**Table 2**). Therefore, understanding the degree and pattern of trait integration among genotypes under different environmental situations might help us to better comprehend plant adaption methods. Co-variation patterns of traits represent important information about plant adaptation strategies selected under different environmental conditions (Laughlin, 2014; Messier et al., 2017). In this study, there was a strong correlation between root traits under low P condition (**Table 2**). The results supported our second hypothesis that the correlation between root traits of vegetable crops was enhanced under low P treatment. This suggests that under low P conditions, vegetable crops could improve co-variation between root characteristics, optimizing fitness. These results are consistent with the theory that trait integration generally increases with environmental stress (Damian et al., 2020; Delhaye et al., 2020).

In this study, A-Pase and carboxylates were correlated with root traits lower (**Table 2**), this is contrary to the findings of Shen et al. (2011), suggesting that vegetable crops can maintain higher phosphatase activity and organic acid content in most combination of root and morphological traits to obtain inorganic P ultimately increasing plant yield. The covariation patterns among root traits were strongly influenced by P supply (Wang et al., 2020). In modern agriculture, plants with high fertilizer inputs are more conducive to P resources (Milla et al., 2015), leading to the decreasing co-variation of traits (Milla et al., 2014; Roucou et al., 2018). Root length and root/shoot ratio have great plasticity. Co-variation of traits with root reduction can potentially result in additional new trait combinations (Laughlin, 2014), to achieve diverse roles and enhance growth in high P environments.

We found that at high P level, vegetable crops depend on the indirect effect of root and carboxylates on crop yield. Though, vegetable crops had increased the direct effect of root at low P level. Meanwhile, mycorrhizal crops use more indirect pathways of influence, such as A-Pase activity-roots and AMF-roots (**Figure 5**). Root morphology and regulation of AMF belong to “P-scavening” strategy, that enhanced the detection range of soil P in a complementary manner (Lambers et al., 2008; Shen et al., 2011). In contrast, the release of P-activated secretions (e.g., carboxylates) is a “P-mining” technique for increasing soil P availability through coordination of exchange and chelation of insoluble phosphorus sources (Hinsinger, 2001; Ryan et al., 2001; Shen et al., 2011). Therefore, the yield of vegetable crops was significantly affected by the “P-mining” strategy, that is contrary to the research results of Lambers et al. (2015), who demonstrated that “P-mining” strategy was more effective when the P content in soil solution was very low and most of P was adsorbed or fixed by soil particles. We suggest that the occurrence of such phenomena may be because of soil P-mining, and vegetable crops can fully distribute their own required P. Under high P condition, the increase of root nutrient cost promotes the secretion of carboxylates, and dominance of “P-mining” strategy in vegetable crops. At low P level, mycorrhizal vegetables showed that both “P-mining” strategy and “P-scavenging” strategy affected vegetable yield. It demonstrates that vegetables use all means within own cost range to absorb more P. Non-mycorrhizal vegetables, on the other hand, lack mycorrhizal pathway absorption, reduce the cost and only root modifications (“P-scavenging” strategy) affect yield. In conclusion, vegetables can improve their ability to take up P under low P conditions by increasing the correlation of their own traits and boost the influencing pathways. Simultaneously, robust and diverse trait combinations would help vegetables produce more stable yield under varying P conditions.

## Conclusions

The present study provides a comprehensive framework for understanding how 12 vegetable species with varying P sensitivity coordinate two shoot traits and 13 root traits to acclimatize to varied P conditions. Our findings revealed that when under P stress, vegetables reduce their own exudation firstly. Non-mycorrhizal plants have relatively stable root traits, whereas solanaceae plants depend more on alterations in root morphological and structural traits. Finally, we revealed that plants were more likely to respond to low P stress by altering their own morphological structure correlation. Meantime, low P stress could significantly improve the direct and indirect ways of mycorrhizal vegetable crops’ root traits axis on shoot biomass, and enhance the direct way of non-mycorrhizal vegetable crops’ root traits axis and reduce the indirect way of root exudates.

## Data availability statement

The raw data supporting the conclusions of this article will be made available by the authors, without undue reservation.

## Author contributions

ZP, RZ and HW performed the experiment, collected the samples and the data. ZP analyzed the data and wrote the first version. HW, QL and JZ edited and revised the manuscript. XW designed the experiment and supported the fund and improved the manuscript. All authors contributed to the article and approved the submitted version.
