# Supplementary material for: Root morphological and physiological traits and arbuscular mycorrhizal fungi shape phosphorus-acquisition strategies of 12 vegetable species
Source: Front Plant Sci. 2023 May 8;14:1150832. doi: 10.3389/fpls.2023.1150832 (PMC10202175; doi:10.3389/fpls.2023.1150832)
Supplement: Supplementary file 1 [file Table_1.docx]

***Supplementary materials***

**Root morphological and physiological traits and arbuscular mycorrhizal fungi shape phosphorus-acquisition strategies of 12 vegetable species**

Zitian Pu^1,2^, Ruifang Zhang^2^, Hong Wang^2^, Qingyun Li^3^, Jianheng Zhang^2,4^ and Xin-Xin Wang^1,2*^

^1^State Key Laboratory of North China Crop Improvement and Regulation, Hebei Agricultural University, Baoding 071001, People's Republic of China

^2^Mountain Area Research Institute, Hebei Agricultural University, Baoding 071001, People's Republic of China

^3^College of Horticulture, Hebei Agricultural University, Baoding071001, People's Republic of China

^4^Key Laboratory of North China Water-Saving Agriculture of Ministry of Agriculture and Rural Affairs, Baoding, Hebei 071001, China

* **Correspondence:**

Corresponding author: sywxx@hebau.edu.cn

**Supporting and table (Tables S1):**

**Table S1** Detailed information of each of the 12 vegetables species used in the experiment.

| Common name | Botanical names | Botanic family | Life form | Abbreviation |
| --- | --- | --- | --- | --- |
| allium | *Allium fistulosum* L. | Lillaceae | perennial | Af |
| garlic | *Allium sativum* L. | Liliaceae | perennial | As |
| cucumber | *Cucumis sativus* L. | Cucurbitaceae | annual | Cs |
| melon | *Citrullus lanatus* | Cucurbitaceae | annual | Cl |
| spinach | *Spinacia oleracea* L. | Chenopodiaceae | annual | So |
| sugar beet | *Beta vulgaris* L. | Chenopodiaceae | biennial | Bv |
| rape | *Brassica chinensis* Linn | Brassicaceae | annual or biennial | Bc |
| lettuce | *Lactuca sativa* Linn | Compositae | annual or biennial | Ls |
| chrysanthemum | *Chrysanthemum coronarium* L. | Compositae | annual | Cc |
| pepper | *Capsicum annuum* | Solanaceae | annual | Ca |
| eggplant | *Solanum melongena* L. | Solanaceae | annual | Sm |
| tomatoes | *Lycopersicon esculentum* Miler | Solanaceae | annual or perennial | Le |
